# Enhanced geothermal systems (EGS) for UN sustainable development goals

**DOI:** 10.1007/s43937-022-00009-7

**Published:** 2022-05-20

**Authors:** D. Chandrasekharam

**Affiliations:** Department of Civil Engineering, Izmir Institute of Technology, 35430 Izmir, Turkey

Energy-water-food nexus, the three interdependent primary requirements are essential for life and the need of the hour by the world. The Covid-19 pandemic has proved that countries that are food independent can survive any stressed conditions. For food security, water is essential. Countries that are self-sufficient with water can overcome situations like what the world experienced in the last 2 years. Energy independent countries can be water secured at a cost that will disturb the environment. But when this energy is green and free from carbon footprint, it will promote sustainable development of a country, providing water and food. This technology, which is in its development stage now, will be the future “energy road” to the countries. High radiogenic granites, omnipresent, with low carbon, and low land footprint, can power the millions. In enhanced geothermal systems or engineered geothermal systems (EGS), as the name implies, a reservoir is created in a hot rock, that is devoid of water, and heat from the rock is mined by circulating water or supercritical carbon dioxide (ScCO_2_) [12]. Three dimensional simulations of EGS systems operated with CO_2_ as injection fluid (heat extraction fluid) instead of water was used by Pruess [13] indicate that gravity effects were found to be strong and capable of inducing preferential flow in the bottom of the thermal reservoir. The advantage of ScCO_2_ is to avoid water based reservoir that inhibits water rock-interaction, resulting in the precipitation of secondary minerals and clogging the fracture openings. This circulating ScCO_2_ is a future promising heat extraction medium with minimum liability on the thermal reservoir.

This heat from the granites is utilized for generating electricity, providing heat to support agriculture, space heating, dehydration and finally providing low-cost freshwater from the sea through desalination technology.

Unlike hydrothermal resources, that are site-specific, EGS has no such restrictions. Starting with traditional hydrofracturing technology, which was established in Soultz-sous-Forets, France and Cooper basin, Australia [9, 11], now EGS is bouncing into a new development of a “closed-loop” system of technology, extracting heat from the granites. This method, unlike the conventional hydrofracturing method, is free from induced seismicity. The closed-loop EGS system is also known as the advanced geothermal systems (AGS) uses a co-axial U-loop in which the working fluid (water or ScCO2) will not enter the rock or flow into the rock fractures [2]. The loop acts as heat exchanger, transferring heat from the rock to the circulating fluid that is collected or flows into a production well. Simulations studies were conducted using varying injection temperatures and varying reservoir temperatures and flow rate [2]. The results show that annual power generation from this system could vary from 2 to 15 GWh with a levelized unit cost of power around US$ 20 to 110 /MWh. Over the years, with technological development this cost could be brought down. The advantage of AGS is, electricity can be generated anywhere on the earth and with minimum or zero induced seismicity that is a cause of concern with hydrofraturing based EGS systems.

I will discuss two cases: one where EGS is sustainable for water-food security and the second case, where EGS is cost-effective and helps the country to support UN SDG. The Former case is Saudi Arabia and the latter one is Turkey. Both the countries have huge EGS resources and how this energy source will help the countries to mitigate climate-related issues and secure food and water are highlighted here.

**Fig. 1:**
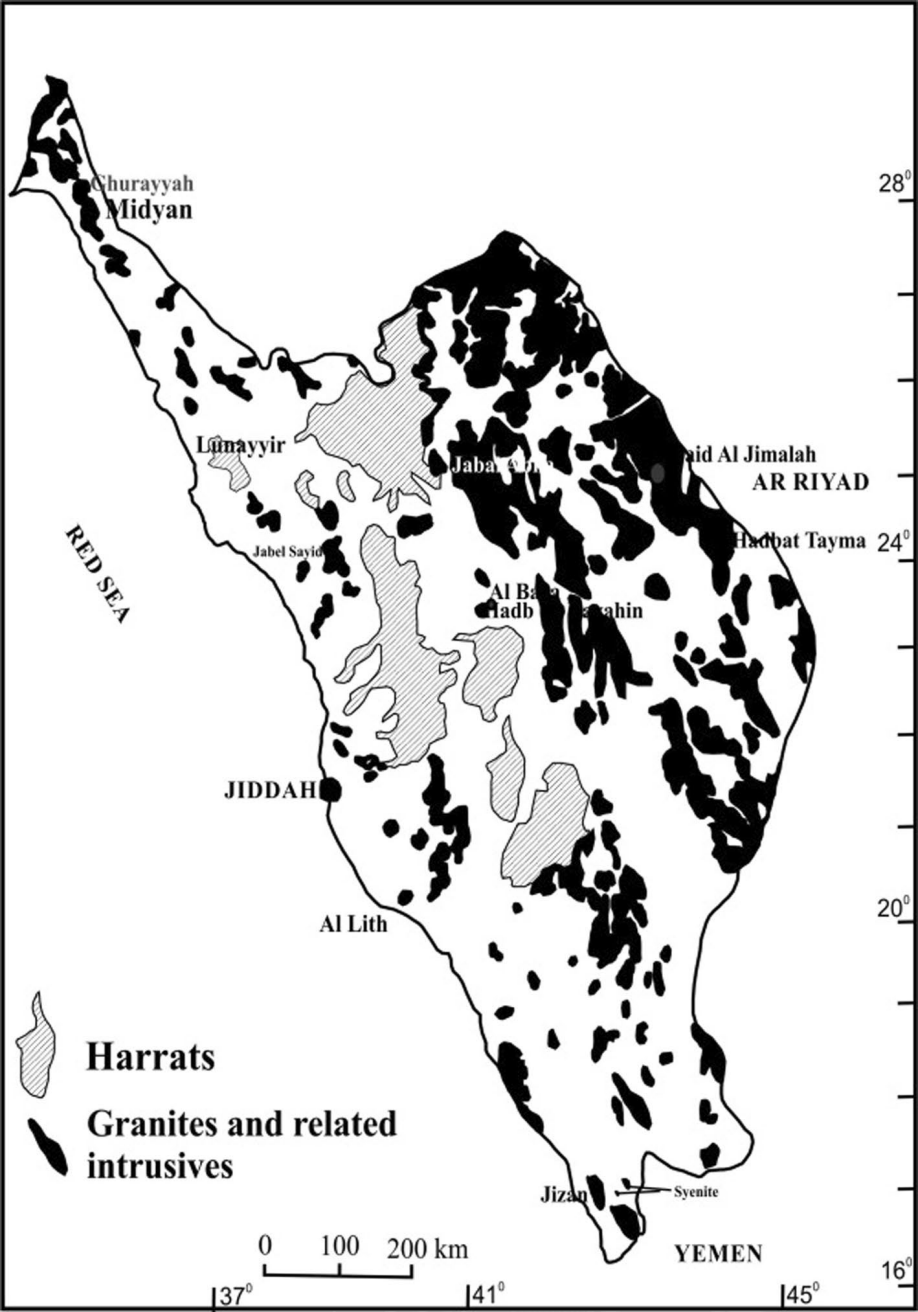
Radiogenic granites with high heat generation capacity, Western Arabian Shield. (adapted from Ref. [6, 7])

The Western Arabian shield hosts numerous granite intrusives (Fig. 1), spread over a cumulative area of 161,467 sq. km, with high concentration of radioactive elements (U, Th, and K) and the radiogenic heat production (RHP) of these granites varies from 5 to 134 µW/m^3^ [6, 7]). The temperatures measured in certain bore-holes along the western margin of the shield gave geothermal gradients exceeding 80 °C/km. Temperature estimates at 2 and 3 km depth gave values of 230–300 °C [6, 7]).

Earlier EGS projects established in Soultz-sous-Forets, France and Cooper basin, Australia [9, 11], estimated that 1 km^3^ of such high radiogenic granites can generate 79 × 10^6^ kWh of electricity. Assuming a 2% recovery of heat from such granites, the power generation capacity of the Midyan granite alone (Fig. 1), located at the NW corner of the shield, exceeds 160 × 10^12^ kWh. Fluid flow and heat transfer models using ANSYS/CFX demonstrate that this amount can be enhanced further to support industrial applications [14]. Saudi Arabia, though energy independent, is a water-stressed country and depends on groundwater for agriculture and domestic needs. The annual groundwater recharge is only 2.4 billion m^3^ while the groundwater extraction is 20 billion m^3^. This additional water is withdrawn from the non-renewable (fossil) aquifer- the Saq-Ram sandstone aquifer. This is not a sustainable solution to meet water demand. Hence the country depends on food imports. Saudi Arabia’s food imports have exceeded 70 million tons and the country has banned wheat production due to a lack of water for irrigation [8]. The country is unable to support per-capita “pita” (bread) production of 88 kg/y. Thus, wheat imports have surged from 1.9 to 3.03 million tonnes. To support other agricultural and domestic water supply, the country depends on 128 desalination plants, supported by fossil fuel-based energy, supplying freshwater at an energy input of 5 kWh/m^3^ (MSF: Multistage Fractionation, [10]). The source of energy being fossil fuel the emissions are around 5.43 kg CO_2_/m^3^ of freshwater generated. EGS (with ScCO_2_ as extraction fluid) has three advantages for Saudi Arabia. It can provide base-load uninterrupted energy, and freshwater (food security) and reduce CO_2_ emissions.

**Fig. 2:**
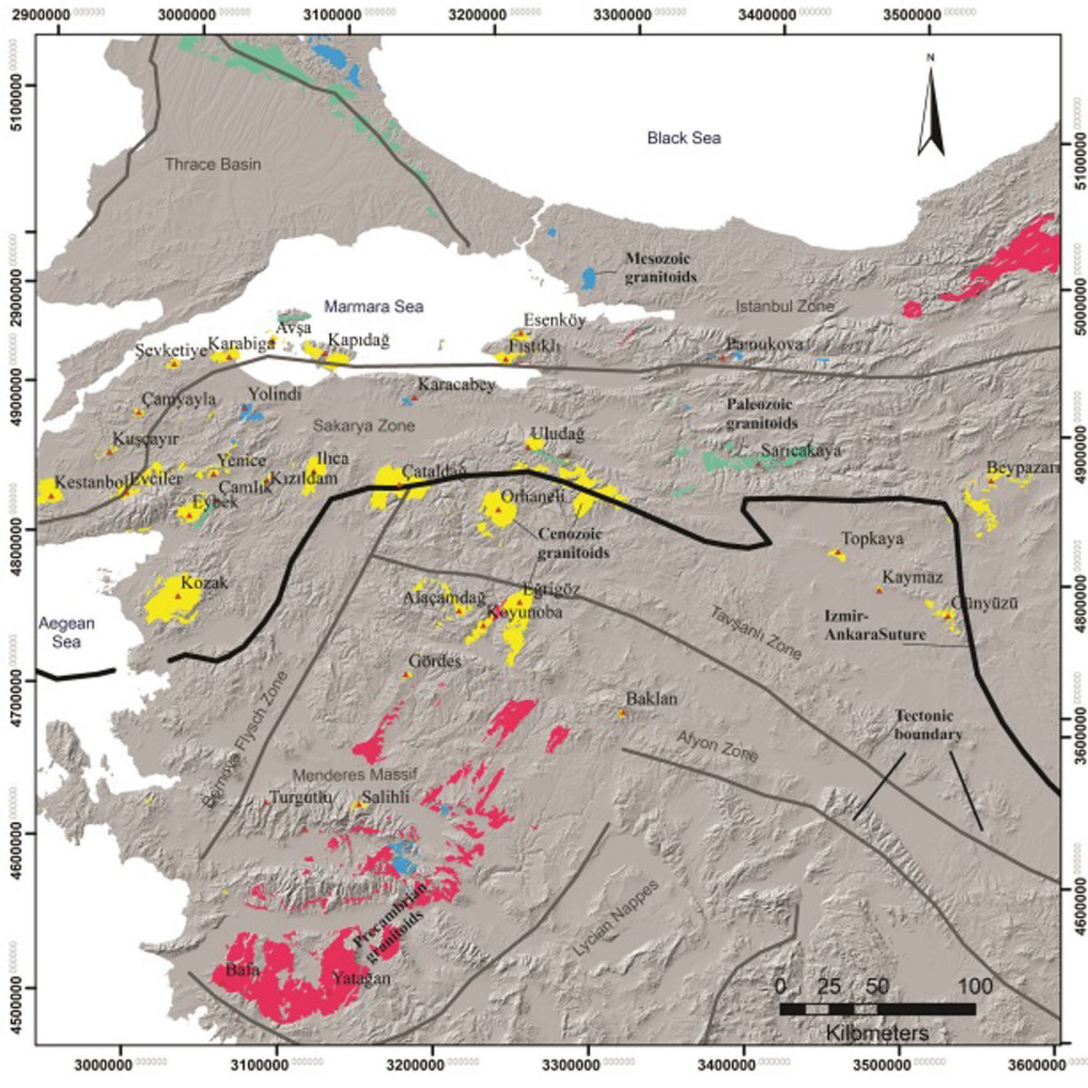
High radiogenic granites of western Anatolia, Turkey. (adapted from Ref. [4])

Turkey’s EGS potential is also huge and similar to Saudi Arabia. High radiogenic Eocene and Miocene granites are spread over a cumulative area of 6910 sq. km along western Anatolia (Fig. 2). Although the RHP of the granites of western Anatolia is lower than that of Saudi Arabian granites, the advantage Turkey has is the disposition of high-temperature isotherm (Curie point temperature-depth) at a very shallow depth (6 to 12 km [1, 3] and a foundered continental and thinned crust due to the Alpine-Himalayan orogeny.

However, the heat flow values of western Anatolian region are very high (~ 154 W/m^2^), which is higher than the heat flow values of the Arabian shield. The reservoir temperatures estimated based on the bottom-hole temperature of drill holes is about 180 °C at 3 km [3]. These granites can generate a minimum amount of 546 × 10^9^ kWh [4]. Turkey has established a strong geothermal culture with its hydrothermal resource, generating 1658 MWe and providing district heating and greenhouse cultivation support. Turkey ranks the third position in the world with its geothermal energy generation. With the development of EGS, Turkey will emerge as a strong country establishing United Nations’ Sustainable development Goals SDG) in another few years. With a high-temperature regime at shallow depth, the cost of drilling will drastically be come down allowing a lower levelised cost of electricity (LOCE). The entire western Anatolian region will be an experimental ground to perform innovative technology related to EGS, like developing loop technology to harness heat, carbon dioxide sequestration, supercritical carbon dioxide fluid circulation to extract heat and develop innovative methods to create fracture networks in the granites at 3 km depth. This provide an opportunity to refine the EGS technology. While the unit cost of energy projected based on earlier EGS projects (Soultz-sous-Forets, France and Cooper basin, Australia) was around 6 to 7 Eurocents, EGS projects in Turkey will provide a realistic unit cost of power in future. Like Solar PV [5], EGS has no carbon footprint and the land footprint is very low. EGS will provide sound energy-water-food security to all the countries and remove the fiscal disparity between the countries and people and lead the world towards a net-zero emissions scenario.
